# Potential Mechanisms Linking Food-Derived MicroRNAs, Gut Microbiota and Intestinal Barrier Functions in the Context of Nutrition and Human Health

**DOI:** 10.3389/fnut.2021.586564

**Published:** 2021-03-09

**Authors:** Ester Díez-Sainz, Silvia Lorente-Cebrián, Paula Aranaz, José I. Riezu-Boj, J. Alfredo Martínez, Fermín I. Milagro

**Affiliations:** ^1^Department of Nutrition, Food Science and Physiology/Center for Nutrition Research, Faculty of Pharmacy and Nutrition, University of Navarra, Pamplona, Spain; ^2^Navarra Institute for Health Research (IdiSNA), Pamplona, Spain; ^3^Centro de Investigación Biomédica en Red Fisiopatología de la Obesidad y Nutrición, Instituto de Salud Carlos III, Madrid, Spain

**Keywords:** miRNAs, xenomiRs, dysbiosis, cross-kingdom regulation, exosomes, intestinal permeability

## Abstract

MicroRNAs (miRNAs) are non-coding single-stranded RNA molecules from 18 to 24 nucleotides that are produced by prokaryote and eukaryote organisms, which play a crucial role in regulating gene expression through binding to their mRNA targets. MiRNAs have acquired special attention for their potential in cross kingdom communication, notably food-derived microRNAs (xenomiRs), which could have an impact on microorganism and mammal physiology. In this review, we mainly aim to deal with new perspectives on: (1) The mechanism by which food-derived xenomiRs (mainly dietary plant xenomiRs) could be incorporated into humans through diet, in a free form, associated with proteins or encapsulated in exosome-like nanoparticles. (2) The impact of dietary plant-derived miRNAs in modulating gut microbiota composition, which in turn, could regulate intestinal barrier permeability and therefore, affect dietary metabolite, postbiotics or food-derived miRNAs uptake efficiency. Individual gut microbiota signature/composition could be also involved in xenomiR uptake efficiency through several mechanisms such us increasing the bioavailability of exosome-like nanoparticles miRNAs. (3) Gut microbiota dysbiosis has been proposed to contribute to disease development by affecting gut epithelial barrier permeability. For his reason, the availability and uptake of dietary plant xenomiRs might depend, among other factors, on this microbiota-related permeability of the intestine. We hypothesize and critically review that xenomiRs-microbiota interaction, which has been scarcely explored yet, could contribute to explain, at least in part, the current disparity of evidences found dealing with dietary miRNA uptake and function in humans. Furthermore, dietary plant xenomiRs could be involved in the establishment of the multiple gut microenvironments, in which microorganism would adapt in order to optimize the resources and thrive in them. Additionally, a particular xenomiR could preferentially accumulate in a specific region of the gastrointestinal tract and participate in the selection and functions of specific gut microbial communities.

## Introduction

MicroRNAs (miRNAs/miRs) are small single-stranded non-coding RNA molecules of ~24 nucleotides in length, discovered in *Caenorhabditis elegans* in 1993 (1, 2). MiRNAs play a key role in post-transcriptional gene expression regulation by their base complementary binding to mRNAs, which promote mRNA degradation or suppress translation (1, 3). MiRNAs participate in several biological functions in animals, such as metabolism (4), immune system development and immune responses (5), proliferation, cell death, differentiation (6) or embryogenesis (7). In plants, miRNAs are attributed a role in processes such as plant development (8) stress responses (9), metabolism or cell signaling (10, 11). Of note, mature plant miRNAs are characterized by a methylation in the 3-terminal nucleotide 2-hydroxyl group, which increases stability by protecting miRNAs from exonuclease degradation (12, 13). In vascular plants, RNA molecules, including miRNAs, could be transferred locally between cells and over long-distances, by the plasmodesmata and the phloem vasculature, which suggest a role for RNA molecules in transmitting information beyond their cell of synthesis (influencing processes such as stress responses or development) (14–16).

Indeed, plant miRNAs have gained special attention in recent years for their potential role as cross-kingdom gene expression regulators, influencing plant interactions with animals and microorganisms, which has been discussed in a number of reports (17–19). Thus, plant miRNAs have been extensively studied for their involvement in plant-microorganism interactions, influencing processes like the arm race co-evolution between host and parasites (20, 21). In animals, diet would be a primary source of phase plant miRNA uptake, subsequently being detected in fluids and tissues of a wide range of species such as mice (22), pigs (23) and humans (24), whereby have been termed as xeno-miRNAs (xenomiRs). The mechanisms through which xenomiRs would penetrate into the bloodstream to eventually reach animal tissues and organs have been reviewed by several authors (17, 25, 26). To sum up,

1. it has been suggested that xenomiRs could be taken up by intestinal epithelial cells through molecules such as transmembrane miRNA carriers or receptor-facilitated endocytosis (17, 25, 26).
2. Alternatively, extracellular vesicles could be incorporated in mammalian cells through mechanism such as phagocytosis, macropinocytosis, clathrin-mediated, caveolin-mediated, and clathrin and caveolin-independent endocytosis (27). Even though the mechanism by which plant extracellular vesicles could be internalized into intestinal epithelial cells is not well-know yet, for example, it has been demonstrated that exosome-like nanoparticles, which contained miRNA, could be incorporated in intestinal epithelial stem cells by micropinocytosis (28).
3. Once xenomiRs are inside intestinal epithelial cells, they could be packaged into microvesicles, which appears to be the main form of transport for at least certain types of xenomiRs, to subsequently be released into the bloodstream (23, 24, 29, 30).
4. Another xenomiR bloodstream transportation mechanism consists in xenomiR association with proteinase K-resistant complexes during digestion or absorption, which has been identified for miR-2911 (31).
5. It has also been postulated that immune system cells could contribute to the xenomiR cross-kingdom transfer by capturing them in the intestinal lumen and releasing them into the bloodstream (25).
6. In addition, it has been suggested that xenomiRs could diffuse paracellularly between gut barrier intercellular spaces (17).

In prokaryotes, in particular bacteria, several small non-coding RNAs (snRNAs) from 50 to 200 nucleotides with post-transcriptional regulatory functions have been also identified. These prokaryotic snRNAs affect bacterial physiology by modulating many processes, such as microorganism virulence and communication, metabolism or stress response (32, 33). A wide range of those snRNAs exert their post-transcriptional effects through base complementary binding to mRNAs targets, being considered functional analogs to eukaryotic miRNAs (32, 34). However, these prokaryotic snRNAs present many differences with eukaryotic miRNAs, regarding their biogenesis, their mRNA binding site, the presentation to mRNA target and/or their mechanism of action, since they can inhibit or enhance mRNA translation, meanwhile eukaryotic miRNAs classically down-regulate gene expression (35, 36). Notably, a subtype of prokaryotic snRNA of similar size to eukaryotic miRNAs has been recently identified. This newly identified snRNA has received several denominations, such as micro-like size sRNAs or microRNA-like molecules, being thus considered as typical miRNAs (35, 37, 38). Indeed, it has been reported that prokaryotic microRNA-like molecules could play an important role as virulence factors (39, 40). For instance, Gu et al. (40) showed that *Salmonella* could take advantage of the machinery of human intestinal epithelial cells to produce microRNA-like molecules as virulence factors, thus promoting bacteria survival and proliferation in mice.

On the other hand, the influence of diet on microbiota composition has been extensively investigated and documented (41–43). Gut microbiota is involved in essential functions for host physiology such as metabolism (through, for example, the fermentation of indigestible carbohydrates and the production of metabolites important for organism homeostasis maintenance) (44, 45), immune system development and function (46), protection against pathogen colonization (47) or intestinal barrier shaping, been involved in their development, reparation and integrity and functionality maintenance (48–51). Additionally, a large number of studies reported that host-derived miRNAs could shape gut microbiota composition through modulation of expression of genes that affect microbe growth (52, 53). In turn, gut microbiota could regulate miRNA expression of host cells, such as intestinal epithelial cells (54–56). Furthermore, some authors pointed out that the bidirectional interaction between host miRNAs and gut microbiota could have an impact on intestinal permeability (52, 54). However, although host fecal miRNAs can modulate the microbiome (53), the role of non-absorbed dietary xenomiRs on microbiota composition modulation by regulating gene expression is scarcely known. Interestingly, it has been postulated that prokaryotic miRNA-like molecules could have an important role in cross-kingdom gene expression regulation, which has been proposed by several authors (35, 57). For example, Shmaryahu et al. (58) predicted pathogenic-bacteria miRNAs that may potentially regulate the expression of human genes involved in several diseases such as diabetes, colon cancer or leukemia. In fact, the authors demonstrated that the prokaryotic miRNAs could down-regulate target gene expression in human cells. The studies carried out by Choi et al. (39) revealed that prokaryotic miRNA-size molecules secreted by periodontal pathogens in vesicles, could enter human cells and down-regulate cytokine expression. However, most of the documented cases of cross-kingdom communication mediated by prokaryote miRNA-like molecules usually refer to pathogenic bacteria and an interaction between non-pathogenic bacteria, such as microbiota, and host cells, mediated by prokaryotic miRNA-like molecules is not well-known. Even so, it has been postulated that not only host miRNAs, but also prokaryotic miRNA-like molecules could also be involved in microbiota-host communication (59, 60). Indeed, and due to the lack of solid evidences regarding the influence of prokaryotic snRNAs, particularly miRNA-like molecules in human physiology, they are out of the scope of the current review.

In this review, we ought to provide new insights on the potential role of the gut microbiota influencing dietary plant xenomiR absorption and to summarize the evidences provided so far regarding the impact that the crosstalk between diet, gut microbiota and mammalian cells could exert on xenomiR absorption/uptake.

## Lack of Consensus on Dietary Xenomir Hypothesis and Potential Explanatory Factors

One of the potential fates of plant-derived xenomiRs incorporated through diet, once they have reached the gastrointestinal tract, is to penetrate into the bloodstream and eventually, to exert a regulation of the expression of target genes in several tissues and organs. However, the specific mechanisms involved in this hypothesis are, to date, still unclear.

Zhang et al. in 2011 (29) reported the presence of rice (*Oryza sativa)* miR-168a in the serum from humans and other animals. This xenomiR could inhibit the expression of the low-density lipoprotein receptor adapter protein 1 (LDLRAP1) in mouse liver, with physiological consequences in the organism, such as the increase of LDL in plasma. After this pioneer study, numerous publications have continued to report the presence of xenomiRs from diverse plants in the serum and tissues of mammals, including humans (22–24, 61, 62). Mlotshwa et al. (63) revealed that oral administered synthetic miR-34a, miR-143 and miR-145, which mimic the characteristics of plant miRNAs, were capable of displaying anti-tumor properties, thus preventing the development and lessen colon cancer progression in mice. Furthermore, Zho et al. (30) discovered that honeysuckle (*Lonicera japonica*) miR-2911, once it had crossed the gastrointestinal barrier and reached the lungs, was able to repress PB2 and NS1 mRNA expression, which encode for two proteins essential for H1N1 Influenza A virus replication, diminishing mortality rate in mice fed with honeysuckle. Subsequently, Chin et al. (64) detected plant miR-159 in human serum and tissues. Their data revealed that, upon oral uptake of synthetic miR-159, *TCF7* gene expression was inhibited, resulting in repression of breast cancer tumor growth in mice. Recent studies are still providing data about the relevance of inter-species gene regulation by plant xenomiRs. Thus, Du et al. (65) showed that *Rhodiola crenulata* small RNA HJT-sRNA-m7 can reach the lungs via intratracheally administration and down-regulate the expression of genes involved in pulmonary fibrosis development in mice, which could culminate in the amelioration of the disease. Moreover, Svecia et al. (66) detected an increase in plant xenomiR levels in the plasma of humans who had taken *Tuscany Sangiovese* grape juice. They also revealed that, in mice, *Tuscany Sangiovese* miRNAs appeared to play a cardioprotective role maintaining the functionality of hearts that had suffered a myocardial infarction.

Despite the huge amount of emerging evidences of the capacity of plant miRNAs from food to be absorbed, travel through the bloodstream and modulate the expression of target genes in mammals, several studies do not fully support the xenomiR cross-kingdom regulation hypothesis due to the inability to detect xenomiRs in serum or tissues. In fact, Zhang et al. (67) questioned technical/methodological issues affecting miRNA detection in publically available databases and alleged that dietary plant miRNAs present in some databases of animal small RNAs could be cross-contaminations during sequencing processes. Some supportive and refuting evidences of the cross-kingdom regulation dietary xenomiR hypothesis have been summarized in Table 1 and have been also extensively summarized by other works (17).

**Table 1:** Some evidences for and against the detection of plant-derived xenomiRs on mammalian fluids and organs.

**Plant xenomiR**	**Source**	**Target mRNA**	**Target tissues/organs**	**Animal model**	**Outcome**	**Concluding remarks**	**References**
**Supportive evidences of the cross-kingdom regulation dietary xenomiR hypothesis**							
miR-168a	Rice	LDLRAP1	Liver	Mice	Decrease LDL removal from mouse plasma	Exogenous plant-food derived miRNAs could regulate gene expression in mammals	(29)
miR-34a miR-143 miR-145	Synthetic plant-mimic miRNAs	Not determined	Intestinal tissue	Apc*^*Min*/+^* mouse model of colon cancer	Reduction in colon tumor burden	Plant-mimic tumor suppressor miRNAs were taken up by the Apc *^*Min*/+^* mouse digestive tract and were functional	(63)
miR-2911	Honeysuckle decoctions and synthetic miR-2911	Influenza A virus mRNA such as PB2 and NS1 mRNAs	Lungs	Mice	Viral replication inhibition	miR-2911 could pass through mouse gastrointestinal tract, be transferred to the bloodstream and lungs and suppress viral infections	(30)
miR-159	Synthetic plant-mimic miRNAs	TCF7	Breast	Mice	Suppression of breast cancer tumor growth	Plant miR-159 could be detected in human serum and breast tumor tissue and inhibit cancer growth in mammals	(64)
HJT-sRNA-m7	*Rhodiola crenulate-*derived HJT-sRNA-m7 agomir	α-SMA, fibronectin, and COL3A1	Lungs	Bleomycin-induced pulmonary fibrosis mouse model	Amelioration of mouse pulmonary fibrosis	HJT-sRNA-m7 may be an effective treatment in anti-pulmonary fibrotic diseases	(65)
Grape miRNAs	SGF	Natriuretic peptides and natriuretic peptide receptors	Cardiac tissue	Mouse model of myocardial infarction	Positive modulation of the cardiac CNP/NPR-B and CNP/ NPR-C pathways	Plant miRNAs could be detected in plasma of healthy humans after drinking SGF and could play a role in the protection of ischemic heart against myocardial infarction	(66)
**Plant xenomiR**	**Source**	**Animal model**	**Outcome**		**Concluding remarks**		**References**
**Refuting evidences of the cross-kingdom regulation dietary xenomiR hypothesis**							
miR-168a	Rice	Mice	Unmeasurable levels of miR-168a in plasma and liver. Not modulation of plasma LDL by miR-168a Not modulation of LDLRAP1 liver levels by miR-168a		Sequence errors or cross-contamination could explain the low number of rice miRNAs detected Nutritional imbalance between test and control groups could explain the Zhang et al. observed increase in plasma LDL The techniques employed to evaluate LDLRAP1 expression could explain the discrepancies with Zhang et al.		(68)
miR-156a miR-159a miR-169a	Western diet containing fruits Custom vegetarian diet Custom soy-enriched diet	Human healthy athletes Mice	Negligible plant miRNAs plasma levels in healthy humans after intake of a western diet containing fruit Negligible plant miRNA plasma or organs (liver, lungs, kidneys and stomach) in mice after intake of custom diets enriched in plant miRNAs		Delivery of xenomiRs via typical dietary ingestion is neither a robust or a frequent mechanism to maintain steady-state xenomiR levels in several animal models		(69)
miR-156 miR-160 miR-166 miR-167 miR-168 miR-172	Plant miRNA-rich substance (a “Silk” fruit and protein shake)	Pigtailed macaques	Detection of some plant miRNAs in plasma but the levels were extremely low and/or the amplification was non-specific		The low levels of miRNAs from plant dietary sources and the variability and non-specificity in amplification did not provide support for the uptake of large proportions of miRNA into the bloodstream		(70)
miR-156a, miR-164a, miR-167a	Corn or corn miRNA extract incorporated in diet	Mice	No detection of corn miRNAs in blood, cecal, fecal or liver samples Significant degradation of corn miRNAs during digestion		No evidence regarding the increase of corn miRNA levels in blood or tissues after supplementation of corn miRNAs in mouse diet that could explain by the early degradation of corn miRNAs in the gastrointestinal tract		(71)
*Viridiplantae* clade miRNAs	EVOO	Healthy humans	Not confidently detection of plant miRNAs in human plasma after acute ingestion of EVOO		It could not be ruled out that the *Viriplantae* miRNA sequences detected in EVOO and plasma samples were the result of a technical artifact due to a library contamination		(72)

*α-SMA, alpha- smooth muscle actin; CNP, C-type natriuretic peptide; COL3A1, collagen type III α 1; EVOO, extra virgin olive oil; LDL, low-density lipoprotein; LDRLAP1, low-density lipoprotein receptor adapter protein 1; NPR, natriuretic peptide receptor; SGF, Sangiovese grape juice; sRNA, small RNA*.

One of the first studies that cast doubt on cross-kingdom uptake of xenomiRs was conducted by Dickinson et al. (68), who unsuccessfully tried to reproduce Zhang's results (29). These authors observed that xenomiRs levels in mouse plasma and liver after ingestion of rice chow were negligible. They also did not detect any change in LDLRAP1 expression in liver, even though LDL levels were increased. So, they postulated that the result obtained by Zhang et al. (29) could arise from sequencing errors, cross-contamination or the use of little sensitive and specific techniques, while the increase in plasma LDL could be a consequence of the impact of diet and not of the xenomiR gene expression modulation (68). However, although their data were controversial, Zhang et al. (29) did not retract their findings, so far.

Afterwards, Snow et al. (69) published a study in which xenomiRs were not detected in the plasma of healthy humans and mice that had been fed with a diet enriched in certain plant miRNAs. On the contrary, Witwer et al. (70) were able to detect a low amount of plant xenomiRs in plasma of macaques fed with plant miRNAs-rich substances, but they concluded that this relatively low xenomiR circulating levels could have been originated in a non-specific amplification. Later, Pastrello et al. (73, 74) retracted their report in which they detected miRNAs from broccoli (*Brassica oleracea*) in the serum of human subjects who had consumed this vegetable, alleging errors in primers design. In the experiments performed by Huang et al. (71) no significant differences were detected in either serum or tissues of mice that had been fed with chow diet supplemented with corn miRNAs. In light of these findings, they suggested that most part of xenomiRs might have been degraded during digestive process and the minimal amount of xenomiRs that could prevail and reach tissues would be insufficient to modulate the expression of endogenous host genes and cause physiological changes. Finally, Micó et al. (72) reported an increase of extra virgin olive oil miRNAs in the plasma of healthy humans but they considered that the negligible levels detected were artifacts resulted from cross-contamination. Analysis of plant miRNAs in human sequencing databases has led many authors to suggest that the underlying causes of xenomiR detection in mammal's body fluids and tissues would be technical artifacts due to cross-contamination during library preparation. The authors conclude that the abundance of xenomiRs in human tissues and fluids would be negligible and there would not be significant differences in terms of xenomiR levels in tissues exposed to food intake, such as the liver, compared to those that have no direct relationship with, like the cerebrospinal fluid (75, 76). Another study demonstrated that miRNA purification columns themselves could be a capture focus for exogenous miRNAs with an unknown origin, many of which appear reflected in human miRNA sequencing databases (77).

Efforts to explain the disparity of evidences for and against xenomiR hypothesis continue monopolizing the core of many investigations. It seems obvious that technical factors such as cross-contaminations, miRNAs purification methods, xenomiR degradation during digestion, and errors during sequencing processes (primer design and non-specific sequencing), could throw into question many of the studies that suggest that xenomiRs could modulate gene expression among different species and kingdoms. Nevertheless, recent reports such as the published by Zhao et al. (78) highlight the need to continue researching in this area and open the door to the exploration of other factors that could shed light and clarify the xenomiRs-host transfer hypothesis. After analyzing an enormous amount of small RNA sequencing data in public databases, Zhao et al. (78) reported that plant miRNAs could reach human fluids and tissues. Unlike previous studies, a remarkably thorough filtering of the sequencing reads was achieved in order to avoid any type of false positives, and cross-contamination was also strongly ruled out (78).

In this way, Zhao et al. (79) recently suggested that one of the factors that could explain the lack of detection of plant miRNAs in animals by certain studies would be the miRNA sequence. Thus, the authors pointed out that not all plant miRNAs could be absorbed, but it would take place a selective absorption reliant on the sequence, in which the high GC content, the shorten length and the motif “CAG” would be determinant in promoting xenomiR uptake (79). Another potential explanatory factor of the variance of xenomiR detection could be intestinal barrier permeability, which will be addressed in the following section.

## Potential Role of Gut Microbiota in the Uptake of Xenomirs in Mammals by Modulating Intestinal Barrier Permeability

Concerning current evidences, we propose that differences in the absorption of plant miRNAs between subjects could be an important factor that could explain, at least in part, the observed variability in the detection of plant miRNAs in mammalian plasma and tissues among different research groups. The permeability of the intestinal barrier could play a major role in the absorption efficiency of xenomiRs and further exploration of its potential role is required. In support of this notion, the studies of Raoof et al. (80) showed an increase of antisense oligonucleotide plasma levels when the epithelial intestinal barrier is leakier. The co-administration of phosphorothioate antisense oligonucleotide ISIS 104838 drug and the permeation enhancer sodium caprate, which promotes changes in intestinal barrier permeability, improved the bioavailability of the drug in dog blood (80, 81). In this context, Yang et al. (61) suggested that the alteration of gastrointestinal barrier permeability could enhance xenomiR uptake and entry to the bloodstream. They observed an increase in diet miRNAs in the bloodstream of mice who had their intestinal barrier integrity altered with cisplatin, suggesting that xenomiR uptake could be enhanced during certain pathologies or chemotherapeutic treatments that alter intestinal barrier integrity (61). Recently, Yang et al. (82) generated mouse models that displayed increased intestinal permeability through the administration of aspirin or anti-CD3 antibodies. In these models, plant-based small RNA uptake was higher as compared to controls. However, their data pointed out that changes in intestinal permeability could only have a significant impact on the absorption of the more stable small RNAs, since the less digestion-resistant RNAs would be degraded (82). Nevertheless, although modulation of intestinal permeability by itself could not have a relevant impact on the bioavailability of the most degradation-sensitive xenomiRs during cooking or digestion, we propose that it could entail special relevance in those cases where xenomiRs were transported in exosome-like extracellular vesicles, since they could be highly resistant to degradation (83, 84) (this issue is further developed and more widely documented in the following section) (section Involvement of Gut Microbiota in Release of Plant miRNAs From Extracellular Vesicles).

Recent studies have highlighted that microbiota could play a key role in maintaining integrity and functionality of intestinal barrier in physiological conditions and during adult lifelong, since it would be involved in determining paracellular permeability through mechanisms such as expression modulation of proteins that are part of tight junctions. Compared to controls, germ-free mice displayed lower paracellular permeability, and transplantation of fecal microbiota from healthy humans led to restoration of paracellular permeability up to levels considered normal, through the decrease of claudin-1 expression and the transient IL-18 production by enterocytes (85).

For this reason, we hypothesize that it would be possible that specific gut microbes could have different effects on intestinal barrier integrity in such a way that a given microorganism could increase permeability while others could be involved in its decrease or have any effect. Thus, there could be an inherent variability as regards of intestinal barrier permeability among different subjects that could depend on the specific composition of gut microbiota. In support of this hypothesis, several authors have reported that colonization of germ-free mice with *Bacteroides thetaiotaomicron* or *Escherichia coli* Nissle 1917 (EcN) led to up-regulation of genes that encode for proteins involved in maintaining adhesion between intestinal epithelial cells, such as small proline-rich protein-2 (sprr2a) and zonula occludens-1 (ZO-1), respectively, which for EcN was proved to result in decreased intestinal permeability when colitis mouse model was treated with this bacterium (86, 87). By contrast, other bacteria such as *Escherichia coli* MG1655 (K12) had no impact on intestinal barrier fortification (87). Results presented in other reports suggest that pathogens, such as enterohemorrhagic *Escherichia coli* (EHEC) O157:H7, enterotoxigenic *Escherichia coli* (ETEC) K88 and *Salmonella typhimurium* SL1344, could compromise intestinal barrier integrity. Infection of epithelial cell monolayers with EHEC O157:H7 promoted claudin-1 and ZO-1 redistribution and further down-regulation of ZO-1 expression, while toxins released upon the infection of epithelial cells with ETEC K88 and *Salmonella typhimurium* SL1344 resulted in disruption of the complexes that maintain adhesion between intestinal epithelial cells (88, 89). Remarkably, the increase in paracellular permeability elicited by these pathogenic bacteria could be ameliorated by certain *Lactobacillus* species (88, 89). It is worth mentioning that non-pathogenic commensal bacteria could also enhance intestinal barrier permeability, like *Escherichia coli* C25, which altered claudin-1 localization increasing intestinal permeability *in vitro* (90).

The influence of microbiota in modulating integrity and functionality of intestinal barrier has been demonstrated in a large bulk of studies showing that gut microbiota dysbiosis could contribute to the alteration of intestinal barrier with notable implications in development and/or progression of diverse pathologies. Cani et al. (91) suggested that microbiota dysbiosis could be the underlying cause of the inflammatory phenotype that characterizes obese and diabetic individuals by disrupting intestinal barrier. They also proposed that an increase of *Bifidobacterium* species abundance would conduce to higher glucagon-like peptide-2 (GLP-2) production which, among other effects, could led to up-regulation of occludin and ZO-1 expression, decreasing intestinal permeability and culminating in a decrease of inflammation (91). For example, hepatic damage caused by cadmium intake could be exacerbated by microbiota dysbiosis. Low-dose intake of this toxic metal for long periods reduced *Akkermansia muciniphila* and increased the relative abundance of Acidithiobacillaceae, Gammaproteobacteria, Methylobacterium, and Rhizobiales in mice. The imbalance in microbe proportion affected occludin, claudin-1 and ZO-1 expression, the down-regulation of which increased intestinal permeability, cadmium uptake and subsequent accumulation in the liver (92). Alteration of intestinal microbiota, specifically the decrease in butyrate-producing gram-positive bacteria and the increase in gram-negative bacteria such as Bacteroidetes, would be the cause of intestinal damage produced after skin burns in mice. The authors proposed that decreased butyrate levels would be primarily responsible for the increase of intestinal permeability and inflammation (93, 94).

It has also been reported that disruption of intestinal barrier integrity on the grounds of a severe physiological stress could be the result of microbiota dysbiosis. Specifically, decrease of Actinobacteria, such as *Bifidobacterium* and *Collinsella*, and increase of Proteobacteria would cause imbalance of several metabolites, like cysteine, thus increasing intestinal permeability (95). The increase of Proteobacteria has also been associated with the leaky gut phenotype that characterizes patients with acute coronary syndrome (96). Likewise, microbiota dysbiosis has been proposed as a mediator of intestinal permeability changes through dysregulation of immune system of children with beta cell autoimmunity at risk for type 1 diabetes (97). In other diseases such as non-alcoholic steatohepatitis, psychiatric disorders or cancer, it has been suggested that the enhancement of intestinal barrier permeability due to microbiota dysbiosis, could aggravate, and even in some cases be a requirement, for their development (98–102).

With all these supportive data, it appears that microbiota could be a key driving force controlling intestinal permeability, which in turn could modulate bioavailability of nutrients and therefore, their presence in blood circulation for potential biological action. It should be noticed that gut microbiota is not static and immutable but it varies between individuals and even within the same individual throughout life, under normal but also pathological states (103). Age, environment, genetics, diet and physiological status could render account for the aforementioned inter- and intra-individual variability observed in humans (103). Intestinal microbiota, together with genetic variability, are considered leading factors determining bioavailability and bioefficacy of plant bioactive compounds, explaining inter-individual variability concerning their digestion, absorption, distribution, metabolism, and secretion (104). For this reason, we propose that intestinal microbiota could be a major factor determining the amount of xenomiRs absorbed through modulation of intestinal barrier permeability under physiological and pathological conditions. We also suggest that divergence regarding specific configuration of microbiota communities among individuals could entail differences in xenomiR uptake efficiency via mechanisms like modulation of gastrointestinal barrier permeability. Some above evidences of the impact of bacteria on intestinal barrier integrity and functionality can be found in Table 2.

**Table 2:** Some evidences of the impact of bacteria on intestinal epithelial barrier functions and integrity.

**Bacteria**	**Model**	**Mediated effect**	**Mechanisms**	**References**
Healthy human gut-derived microbiota transplantation	Germ-free mice	Colon mucus layer fortification Physiological colonic paracellular permeability levels establishment	Decreased claudin-1 expression Transiently increased epithelial IL-18	(85)
*Bacteroides thetaiotaomicron*	Germ-free mice	Potential role on intestinal epithelial barrier fortification (not determined)	Sprr2a mRNA increase	(86)
*Escherichia coli* Nissle 1917	Germ-free mice Colitis mouse model	Colonic epithelial permeability reduction in colitis mouse model	Up-regulation of ZO-1 mRNA in intestinal epithelial cells of germ-free mice and colitis mouse model	(87)
*Lactobacillus rhamnosus* GG	MDCK-I and T84 polarized intestinal epithelial cell monolayers	Protection against induced damage of epithelial monolayer barrier by EHECO157:H7	Inhibition of tight junction redistribution Inhibition of EHEC O157:H7 mediated ZO-1 down-regulation Inhibition of cytoskeleton rearrangements Attenuation of TER reduction	(88)
*Lactobacillus fructosus* C2	Polarized Caco-2 cells (enterocytes)	Decreased of pathogenic bacteria (ETEC K88 and *Salmonella typhimurium* SL1344) permeability increase	IL-8 secretion reduction Tight junction protection ERK and JNK phosphorylation inhibition Attenuation of TER decrease	(89)
*Escherichia coli* C25	MDCK-I and T84 polarized intestinal epithelial cell monolayers	Physiological and structural changes in epithelial barrier function Permeability increase (drop in decrease in TER)	NF-κB activation and IL-8 secretion in T84 monolayers Cytoskeleton rearrangements in T84 monolayers Claudin-1 localization alteration in T84 monolayers ZO-1 distribution alteration and down-regulation in MDCK-1 monolayers	(90)

*EHEC, enterohemorrhagic Escherichia coli; ERK, extracellular signal-regulated kinase; ETEC, enterotoxigenic Escherichia coli; IL, interleukin; JNK, c-Jun N-terminal kinase; sprr2a, small proline-rich protein-2; TER, transepithelial electrical resistance; ZO-1, zonula occludens-1*.

## Involvement of Gut Microbiota in Release of Plant miRNAs From Extracellular Vesicles

Extracellular vesicles have been discovered in both prokaryotic and eukaryotic organisms, including bacteria, archaea, fungi, plants, and mammals. They carry several types of bioactive molecules and thus, they are key factors in intercellular communications (105). In bacteria, extracellular vesicles range from 20 to 400 nm in diameter and they have been involved in bacterial functions such as biofilm formation, competition, antibiotic resistance or horizontal gene transfer (106). Extracellular vesicles of bacterial origin are not only involved in bacteria-bacteria communication, but also in bacteria-host cell communication, being able to elicit phenotypic changes in host cells altering their function with both detrimental and beneficial actions (107–109). Characterization studies of their cargo showed that they can contain several types of molecules, including structural and soluble proteins such as enzymes, secondary metabolites, virulence factors, glycolipids, lipopolysaccharides, bacterial antigens and a wide range of nucleic acids, including DNA and microRNA-like molecules, which have been extensively summarized in other reviews (110–112). Remarkably, gut microbiota can release extracellular vesicles that can influence host physiology, playing part in processes such as digestion (by carrying digestive enzymes), intestinal permeability regulation, metabolism or gut immunity (113–115).

In mammals, extracellular vesicles range in size from 50 to 100 nm in diameter and they modulate system biology as tissue repair, coagulation or embryonic development. Mammalian extracellular vesicles are also involved in disease progression, such as cancer, neurodegenerative and/or metabolic diseases (33, 116–118). The cargo of mammalian extracellular vesicles include lipids (being enriched in lipids such as cholesterol and sphingolipids), cytokines, proteins (in particular plasmatic and cytosolic proteins), nucleic acids (DNA and RNA species, such as miRNAs and mRNAs), amino acids, fatty acids and sugars, and they largely reflect the parental cell content (117, 119, 120). Mammalian extracellular vesicles are classified in microvesicles, exosomes and apoptotic bodies, which differ in their biogenesis and size (121). Contrary to microvesicles and exosomes, apoptotic bodies can contain organelles and chromatin, since they are released from cells undergoing programmed cell death (120). Notably, host extracellular vesicles could also influence gut microbiota, occurring a bidirectional communication mediated by microbiota and host extracellular vesicles (122).

In plants, extracellular vesicles range from 400 to 1,000 nm, depending on the species, and they have similar features (i.e., cargo, morphology and secretion) as mammalian exosomes (110, 123). Plant extracellular vesicles, frequently referred as “exosome-like” nanovesicles, contain lipids, proteins, metabolites and nucleic acids, including smalls RNAs (110, 123, 124). Interestingly, the lipids that constitute the nanovesicle surface confer specificity of uptake by different cell types (125). Plant extracellular vesicles are also known to contain antimicrobial compounds, including defense-signaling lipids and defense-related proteins, and they are involved in functions such as defense against pathogens, immune response, growth and development or symbiosis (124, 126). Plant exosome-like nanovesicles could have an important role in inter-kingdom communication. For example, they could be uptaken by gut microbiota or mammalian cells and their cargo, such as xenomiRs, might have a relevant impact, as it will be further detailed in the following sections (83, 125).

Plant cells can secrete miRNAs to the extracellular medium, as a part of ribonucleoproteins and/or encapsulated in vesicles of different origins (14), which has been reviewed elsewhere (26, 127). It has been observed that a wide range of edible plant can release exosome-like nanovesicles that yield biological effect in mammals. It has also been proposed that plant exosome-like nanovesicles could be an efficient mechanism of transporting bioactive compounds since they provide resistance to degradation, which together with its biocompatibility, led them to even apply as drug delivery system to treat diseases (128–131). The characterization of plant exosome-like nanovesicles has revealed that, among the small RNA cargo, they are enriched in miRNAs, which are selectively loaded in them (128, 132, 133).

Of note, a study carried out by Philip et al. (134) suggested that plant xenomiR bioavailability could be enhanced by processes that promote cell wall disintegration, such as cooking. The authors observed that miRNA levels were higher in cooked beans and brown rice compared to raw controls, and that cooking food promotes the miRNA release into the cooking water. Interestingly, several studies have documented that certain bacteria belonging to Firmicutes and Bacteroides phyla (such as *Ruminococcus champanellensis, Bacteroides intestinalis* or *Bacteroides thetaiotaomicron*), which are part of the human gut microbial community, are able to degrade cellulose, hemicellulose and pectins, major components of the cell wall (135–139). In fact, it has been attributed a role for gut microbiota in enhancing bioaccesibility of fiber-encapsulated nutrients, allowing its release through enzymatic activities capable of fermenting plant-cell wall component, which would be crucial for intestinal nutrient absorption (140, 141).

Furthermore, some studies suggested that some gut microbiota bacteria could hydrolyse lipids via lipase enzymes (142). Indeed, specific bacteria from bovine raw milk possess phospholipolytic activity capable of disrupting milk fat globule membranes, whose main constituents are phospholipids, as in cellular membranes and extracellular vesicles (128, 143, 144). Remarkably, it has been proposed that gut microbiota could also utilize lipolytic and phospholipolytic enzymes to digest milk fat, which in turn might led to changes in gut microbiome (145), which should be deeply explored and confirmed in further studies. Indeed, ongoing studies are exploring the biochemical pathway by which gut microbiota could digest milk fat globules through lipolytic and phospholipolytic enzymes activities (146) to further study the relevance of this newly proposed hypothesis.

Although this field of research has not been explored in depth yet, together, the summarized findings have led us to propose the following hypothesis: gut microbiota could contribute to degrade milk fat globule membrane lipids, as well as cellulose, hemicellulose and pectin fibers. In a similar manner, specific gut microbiota composition could be able to hydrolyse envelope lipids of miRNA-containing plant extracellular vesicles and/or cellulose, hemicellulose and pectin fibers (that might have become attached to plant extracellular vesicles during exocytosis and come out across the cell wall), which might enhance the amount of xenomiRs released in the gastrointestinal tract. Therefore, it should be explored if gut microbiota could play a relevant role in determining the bioaccessibility and bioavailability of plant miRNA encapsulated in extracellular vesicles, through degradation of extracellular vesicle envelope components.

Alto, it is worth to underline that plant extracellular vesicles can directly interact with target cells and release their cargo through several mechanisms such as macropinocytosis or endocytosis (28, 147). Thus, one of the issues that pose the above proposed hypothesis on the role of gut microbiota eliciting the release of miRNA from plant extracellular vesicles, is whether (a) it could be a complementary or facilitating mechanism to promote *in vivo* capture of plant extracellular vesicles, or on the contrary, (b) it could interfere with the suggested selective capture of plant nanovesicles that might depend on the specific lipid membrane composition (125). It would be also worthy to address whether the hypothetical microbiota-dependent plant xenomiR release could entail special relevance in those cases in which plant xenomiRs must be available to exert the documented non-canonical function of binding to cell surface receptor and thus, to modulate cellular signaling pathways (148, 149).

Another issue in which it should be important to dig into consists on determining whether inter-individual differences in gut microbiota composition between healthy subjects or dysbiosis associated to some metabolic diseases like obesity (150, 151) could lead to dissimilarities on the efficiency of the proposed plant xenomiR extracellular vesicles release mechanism. Finally, we hypothesize that gut microbiota could play a major role determining plant xenomiR intestinal absorption rate through the modulation of both miRNA extracellular vesicles release and intestinal barrier permeability, which might partly been explained by the disparity of results regarding the detection of plant xenomiRs in mammals and the eventual veracity of the still controversial plant xenomiR cross-kingdom regulation hypothesis.

## Insight on the Impact of Dietary Plant miRNAs on Intestinal Barrier Function and Permeability

Some studies have suggested that most dietary plant xenomiRs would be degraded during digestive processes and the amount that could reach host cells might not be enough to exert cross-kingdom gene expression regulation (71, 82, 152). However, substantial amount of reports has unveiled that plant xenomiRs would be able to withstand harsh conditions of processing, cooking and digestion (22, 23, 134), and thus, could reach a functionally relevant copy number *per* cell that might surpass a minimum threshold to effectively modulate endogenous host gene expression (153). Within this context, it has been well-documented that factors such as plant miRNA GC content (30), 3'terminal nucleotide 2'-O-metylation of plant miRNAs (13, 29), RNA-binding proteins (154, 155) and especially the packaging into exosome-like nanovesicles (83, 84, 147, 156, 157), are responsible for plant xenomiR stability, greatly endowing them with the capacity to remain unaltered and reach the gastrointestinal tract, to potentially modulate host gene expression.

Another aim of this review is to provide new insights into the potential role of plant xenomiRs in modulating gut barrier function, by interacting with gut microbiota, intestinal epithelial cells and intestinal immune system. The description of the global plant xenomiR effects, once they are absorbed and travel through the bloodstream reaching specific mammalian tissues and organs, goes beyond of the scope of this review and can be found summarized in recent works (17, 127, 158).

### xenomiR Influence on Intestinal Permeability Through Modulation of Microbiota Gene Expression

It is well-established that plant small RNAs can be captured by bacteria and fungus plant parasites and down-regulate the expression of genes related to their invasion capacity (21, 159–161). As an example, several studies have found that *Arabidopsis thaliana* can secrete small RNAs encapsulated in exosome-like extracellular vesicles which have the ability to modulate expression of genes involved in virulence of the fungus *Botrytis cinerea* and the bacterium *Pseudomonas syringae* pv. *tomato* DC3000 (162, 163).

The broad impact of xenomiRs in shaping gut microbiota has also been suggested in some studies. Zhou et al. (164) reported that dietary bovine milk exosomes could modulate gut microbiota composition in mice. They observed that dietary milk exosomes were able to exert a significant impact on the proportion of cecum microbes, stimulating the growth of certain bacteria such as Tenericutes, Firmicutes, and Lachnospiraceae. The authors suggest that dietary milk exosomes-derived miRNAs could be the effectors of the microbiota shaping (164). Similarly, a recent study carried out by Teng et al. (125) establishes a direct causal relationship between plant xenomiRs and alteration of gut microbiota composition and spatial distribution, with physiological consequences on the host. The authors determined that ginger-derived exosome-like nanoparticles could be selectively taken up by *Lactobacillus rhamnosus*, and the diverse miRNAs carried inside these vesicles could regulate the expression of several bacterial genes improving colitis in mice. For example, gma-miR-396e inhibited the expression of the transcriptional repressor LexA promoting *Lactobacillus rhamnosus* growth, which started a cascade control through metabolites, having an impact on the growth of other bacteria species like *Escherichia coli*. Other plant miRNAs like ath-miR-167a could have an influence in the localization of *Lactobacillus rhamnosus* via regulation of SpaC expression, which is necessary for translocation to the bloodstream, promoting the permanence of the bacteria in the mucosa surface. Furthermore, mdo-miR-7267-3p could down-regulate ycnE expression, increasing indole-3-carboxaldehyde (IA3) production and resulting in reduced gut permeability and enhancement integrity (125).

In view of the fact that certain plant xenomiRs could decrease intestinal permeability through regulation of microbiota gene expression, we suggest that plant xenomiRs could also act in the opposite direction, shaping gut microbiota composition and metabolite production in a way that could promote the increase of gut barrier permeability, which could potentially enhance xenomiR, postbiotics and nutrient absorption.

### Role of xenomiRs on Intestinal Permeability Through Modulation of Intestinal Epithelial Cell Functions

Several studies have unveiled that plant exosome-like nanovesicles could target intestinal epithelial cells in mammals and exert biological functions under physiological and pathological conditions. Thus, three independent studies reported that orally delivered fruit-derived exosome-like nanovesicles could be internalized in intestinal stem cells from mice and rats and trigger Wnt/β-catenin signaling pathway, thus enhancing cell proliferation, which contributes to intestinal barrier integrity and homeostasis (28, 83, 156). Interestingly, it has also been shown that some of these nanovesicles, such as grape-exosome-like nanovesicles, could control colitis progression in mice by activating intestinal stem cell Wnt/β-catenin pathway (28). Likewise, results provided by Zhang et al. (84) pointed out that orally administered ginger exosome-like nanoparticles targeted colon intestinal epithelial cells of mice with colitis. There, they promoted cell proliferation, inhibited apoptosis and enhanced the expression of adherent junction proteins like E-cadherin and desmoglein, which, among other factors, led to an improvement of intestinal barrier function and integrity and the attenuation of disease severity. Interestingly, in a colorectal cancer mouse model, ginger exosome-like nanoparticles displayed the opposite effect, inhibiting proliferation and promoting apoptosis of intestinal epithelial cells, which reduced tumorigenesis (84).

In some of the aforementioned studies, the characterization of plant exosome-like nanovesicles cargo revealed that they were enriched for diverse miRNAs, and their bioinformatic analysis revealed that some of them could potentially regulate mammalian gene expression. However, the existence of a direct causal relationship between xenomiRs and the above described extracellular vesicle effects on intestinal epithelial cells was not explored or directly linked (28, 83, 84). Indeed, it could be possible that those extracellular vesicle effects might be dependent on other (complementary) non-xenomiR-related mechanisms.

Interestingly, the direct impact of plant xenomiRs on intestinal epithelial cell gene expression modulation has not been deeply addressed yet. Nevertheless, some studies have proposed that specific xenomiRs could have an impact on intestinal transporter expression or enterocyte proliferation (153, 165, 166), which suggests a potential role for xenomiRs in the modulation of the function of intestinal cells, including molecule absorption or intestinal barrier integrity and functionality. In this context, Fujita et al. (165) reported that Caco-2 cells treated with apple exosome-like nanovesicles displayed changes in mRNA expression of several intestinal transporters including the organic-anion-transporting polypeptide (OATP2B1), whose mRNA and protein expression, along with its activity, decreased, probably by a miRNA-mediated mechanism, which potentially could modulate absorption of orally administered compounds *in vivo* (165). It has also been reported that synthetic plant miR-167e-5p regulated the expression of Wnt/β-catenin signaling pathway key molecules, such as β-catenin and c-Myc, negatively impacting on enterocyte proliferation *in vitro* (166). Similar results were also observed for plant miR-156 treatment *in vitro* and *in vivo*. Exogenous administration of plant miR-156, as a synthetic form or as a part of the diet, suppressed enterocyte proliferation and affected intestine development in mice. These effects were mediated by specific miRNAs targeting mRNA that drive the expression of Wnt10b, a Wnt/β-catenin pathway component, and by promoting phosphorylation and degradation of β-catenin proteins (153).

It is well-known that Wnt/β-catenin cascade plays a crucial role in controlling epithelial cell proliferation and differentiation and it is essential for the regulation of intestinal homeostasis by contributing to the maintenance of gut barrier integrity and functionality (167, 168). Wnt/β-catenin pathway activation also regulates cell regeneration after epithelial damage by enhancing epithelial cell proliferation (169–171). In this context, a correlation has been described between decreased intestinal cell proliferation and increase of intestinal barrier permeability, which could even aggravate the progression of certain pathologies (172, 173). Wnt/β-catenin pathway could not only influence intestinal barrier permeability by controlling proliferation, but it could also impact on intestinal permeability directly promoting expression of proteins that are part of tight junction, such as claudin-1 and 2 (174–177).

Whether plant xenomiRs could have an impact on intestinal permeability by modulating enterocyte gene expression has not been confirmed yet. Nevertheless, since certain plant xenomiRs could affect the expression of enterocyte transporters and activate Wnt/β-catenin pathway (153, 165, 166), it is plausible that, after being taken up by epithelial cells, xenomiRs could potentially act as regulators of intestinal barrier permeability through their ability to specifically regulate intestinal epithelial mRNAs. Subsequently, this might influence nutrient absorption and even the efficacy of xenomiR absorption as an auto-regulatory loop. It is clear that more evidence is needed to prove the influence of xenomiRs on intestinal permeability through regulation of intestinal cell gene expression and to determine if intestinal permeability could be differently modulated depending on the type of xenomiR.

### Relevance of xenomiRs on Intestinal Permeability Through Modulation of Immune System Functions

Growing evidence suggests that plant exosome-like nanovesicles could be taken up by immune system cells and exert immunomodulatory effects. Chen et al. (178) reported that ginger rhizome exosome-like nanovesicles were taken up by macrophages *in vitro* and repressed inflammasome activation, whose activity has been associated with increased intestinal permeability and progression of diseases like obesity (179, 180). Recently, a study carried out by Cao et al. showed that ginseng-derived nanoparticles led to a decrease of melanoma tumor growth in mice by targeting macrophages and suppressing M2 phenotype switching (181).

Furthermore, several studies have directly confirmed that plant-exosome-like nanoparticles have also biological effects on gut immune system. For example, it has been revealed that plant edible exosome-nanoparticles from several fruits and vegetables, such as ginger, grapefruit or carrot, were selectively taken up by intestinal macrophages and exerted anti-oxidant and anti-inflammatory activities (83, 84, 147). For instance, grapefruit and ginger exosome-nanoparticles were able to reduce inflammation and ameliorated induced colitis in mice (84, 147). Interestingly, many molecules and cytokines whose expression has been shown to be modulated by plant edible nanoparticles in intestinal macrophages, such as IL-6, IL-10, IL-22, heme oxygenase-1 (HO-1), nuclear factor (erythroid-derived 2)-like 2 (Nrf2) TNF-α or IL-1β, are able to regulate intestinal permeability (83, 84, 147, 182–188). Moreover, it has been reported that plant edible nanoparticles could be taken up by other intestinal immune cells, in addition to macrophages, as demonstrated by a study conducted by Deng et al. (189). This study showed that the oral administration of broccoli nanoparticles targeted intestinal dendritic cells and activated adenosine monophosphate-activated protein kinase (AMPK), which induced a tolerogenic dendritic phenotype associated with reduced inflammation and colitis protection (189).

Under these premises, it seems clear that plant exosome-like nanoparticles could enter immune system cells, including gut resident immune cells, and modulate immune function. However, the bioactive molecules responsible for the immunomodulatory effects of plant exosome-like nanoparticles have not been deeply studied yet. Interestingly, some authors suggest that lipids of nanoparticles could be responsible for the observed effects on inflammasome inhibition of macrophages in culture or on the suppression of M2 phenotype of macrophages in melanoma cancer (in the last case, the authors suggest that proteins of nanoparticles may also be involved) (178, 181). Interestingly, characterization of the cargo of edible exosome-like nanoparticles taken up by intestinal macrophages revealed that they are highly enriched in miRNAs, as previously described for intestinal epithelial cells (83, 84, 147). However, the direct implication of xenomiRs on the eventual regulation of immune-modulatory functions has not been explored yet. Indeed, it has not been ruled out that proteins, lipids or other bioactive components present in these nanovesicles, such as naringenin in grape exosome-like nanovesicles or 6-gingerol and 6-shogaol in ginger exosome-like nanoparticles, could be the effectors (83, 84, 147). Another study suggested that sulforaphane was the effector of tolerogenic intestinal dendritic cell induction (189).

Of note, several studies have confirmed that xenomiRs could shape mammalian immune system. It has been recently reported that milk-derived extracellular vesicles, through immune-related miRNAs, could enhance intestinal immune system in mice by stimulating IgA and sIgA production, which play a role in maintaining intestinal barrier integrity and function. The authors also suggest that the modulation of microbiota composition and their metabolites upon the milk-derived exosome treatment could contribute to the shaping of intestinal immune system (190). Notably, there is accumulative evidence showing that milk-derived extracellular vesicles contain plant miRNAs (191, 192), suggesting that eventually, immune system shaping might be attributed to xenomiRs. Indeed, it has been pointed out that plant miRNAs acquired through diet could be captured by human mammary glands, packaged into exosomes and transmitted through milk to infants (193). However, further research is required to determine if plant miRNAs could play part in the immunomodulatory effects assigned so far to milk-derived exosomes (194–197).

Furthermore, Zhang et al. via computational approaches, predicted *Arabidopsis thaliana* miRNA target genes in humans and their findings revealed that plant xenomiRs could regulate immune system functions, such as leukocyte and lymphocyte activation and inflammation (198). Aquilano et al. provided experimental evidence on the ability of plant miR-159a and miR-156c from nut exosome-like nanovesicles to display anti-inflammatory effects by down-regulating TNF receptor superfamily member 1a (Tnfrsf1a) mRNA expression in macrophages and adipocytes *in vitro*, which impacted negatively on TNF-α signaling pathway (199). Remarkably, the treatment of diet-induced obese mice with nut exosome-like nanovesicles reduced adipose tissue pro-inflammatory cytokine levels (including TNF-α mRNA) and improved the metabolic profile (199). Several studies have described that intestinal immune system and TNF-α signaling pathway are involved in the increased intestinal permeability observed during obesity by the overexpression of pro-inflammatory cytokines (such as TNF-α) in the intestine, which contribute to the restructuration of the proteins that are part of tight junctions. The dysfunction of intestinal barrier is implicated in the worsening of the disease since it exacerbates endotoxemia and increases circulating pro-inflammatory cytokine levels, enhancing chronic inflammation (187, 200, 201). Since plant miRNAs could suppress TNF-α signaling pathway, it would be interesting to explore if they could modulate intestinal immune system and gut barrier permeability, and if this effect would eventually contribute to the reduction of inflammation in metabolic tissues during obesity development.

Finally, Cavalieri et al. (149) demonstrated that a large range of miRNAs from diverse plant species, through a sequence independent mechanism, could act as Toll-like receptor 3 (TLR3) ligands in dendritic cells, attenuating the innate immune response induced upon an inflammatory stimulus *in vitro*. The authors also found that plant xenomiRs, via impairment of TLR3 signaling pathway, were able to reduce inflammation and to improve mice autoimmune encephalomyelitis prognosis (149). It should be noted that there is a strong connection between increased intestinal permeability and autoimmune encephalomyelitis onset and progression (202) and that dendritic cells could contribute to intestinal barrier dysfunction in inflammatory diseases (203). These data suggest the potential impact that xenomiR-induced dendritic tolerogenic phenotype induction could have on gut barrier permeability and protection against autoimmune diseases.

It is worthy of note that there are some evidences that plant xenomiRs could shape immune system through gut microbiota modulation, leading to changes in intestinal barrier permeability. In this context, Tent et al. reported that plant mdo-miR-7267-3p repressed ycnE expression in *Lactobacillus rhamnosus*, upregulating the production of I3A and leading to increased IL-22 production [by activating aryl hydrocarbon receptor (AHR) pathway in lymphocytes], which in turn strengthened gut barrier and improved colitis in mice (125).

Table 3 summarizes most relevant evidences of plant-dietary xenomiRs interaction with mammalian cells. Together, the evidence presented above suggests a potential role for plant xenomiRs in the modulation of cells linked to immune system, including intestinal immunity. It seems that plant xenomiRs might contribute to intestinal barrier integrity and function through mechanisms mediated directly by immune system cells (for example, by means of gene expression regulation or receptor binding and signaling pathway modulation) or acting indirectly on the immune system through gut microbiota. However, further studies are needed to confirm this hypothesis.

**Table 3:** Some supporting *in vitro* and *in vivo* evidences of the interaction of plant-dietary xenomiRs with mammalian cells.

**Plant xenomiR**	**Source**	**Target mRNA/molecules**	**Model**	**Mediated effect**	**References**
miR-167e-5p	Synthetic methylated plant miRNA mimic	Wnt/ β- catenin related genes (such as β- catenin, c-Myc and PCNA mRNA)	IPEC-J2 and Caco-2 cells (enterocytes)	Proliferation suppression	(166)
miR-156	Synthetic methylated plant miR156 mimic Maize diet	Wnt10b mRNA)	IPEC-J2 cells (enterocytes) Mice	IPC-J2 proliferation suppression Intestine development regulation	(153)
miR-159 miR-156c	Exosome-like nanovesicles isolated from commercially available edible dried nuts Small RNAs isolated from dried nuts Synthetic methylated plant miR159 and miR156c mimics	Tnfrsf1a mRNA	3T3-L1 and T37i cells (adipocytes) RAW 264.7 (macrophages) and Human type 1 macrophages Mice	Reduction of inflammatory markers in hypertrophic and TNF-α-treated adipocytes and macrophages by down-regulation of TNF-α signaling pathway Enhancement of glucose uptake in adipocytes Suppression of inflammation and improvement of metabolic profile in obese mice treated with exosome-like nut nanovesicles	(199)
FvmiR-168	miRNA-rich small RNA extracts from a wide range of plants	Interaction with TLR3 receptor	Dendritic cells Mouse model of multiple sclerosis (EAE)	Modification of dendritic cells ability to respond to inflammatory stimulus Reduction of inflammation and EAE onset and severity	(149)

*EAE, experimental autoimmune encephalomyelitis; FvmiR-168, Fragaria vesca miR-168; PCNA, proliferating cell nuclear antigen; TLR3, toll-like receptor-3; TNF receptor superfamily member 1a (Tnfrsf1a)*.

## Conclusions and Future Directions

The current lack of consensus regarding the veracity of the plant-derived xenomiR hypothesis highlights the need to thoroughly identify the factors underlying the high variability of results obtained in the different studies, including optimizing technical methods. One of the factors that might potentially contribute to explain the lack of detection of plant miRNAs in animals by certain studies could be intestinal permeability, since it could play a relevant role in xenomiR absorption. In parallel, there is a strong support for the crucial role of gut microbiota controlling intestinal barrier integrity and function, standing as an important modulator of intestinal permeability. Remarkably, gut microbiota composition varies in an intrinsic manner between healthy individuals (inter-individual variability) and it is well-known that changes in gut microbiota composition and functionality could promote variability in the uptake of plant bioactive compounds through intestinal barrier modulation. Therefore, it would be necessary to explore whether the differences in microbiota composition between healthy subjects could lead to differences in intestinal permeability and whether these normal dissimilarities would be significant enough to endorse variability in xenomiR absorption rate. On the other hand, since gut microbiota dysbiosis could contribute to the onset and progression of a wide range of diseases by affecting intestinal barrier permeability, the link between the alteration of intestinal permeability (as result of gut microbiota specific composition in a pathological context/dysbiosis) and xenomiR bioavailability should also be comprehensively addressed in future researches.

**Figure 1:**
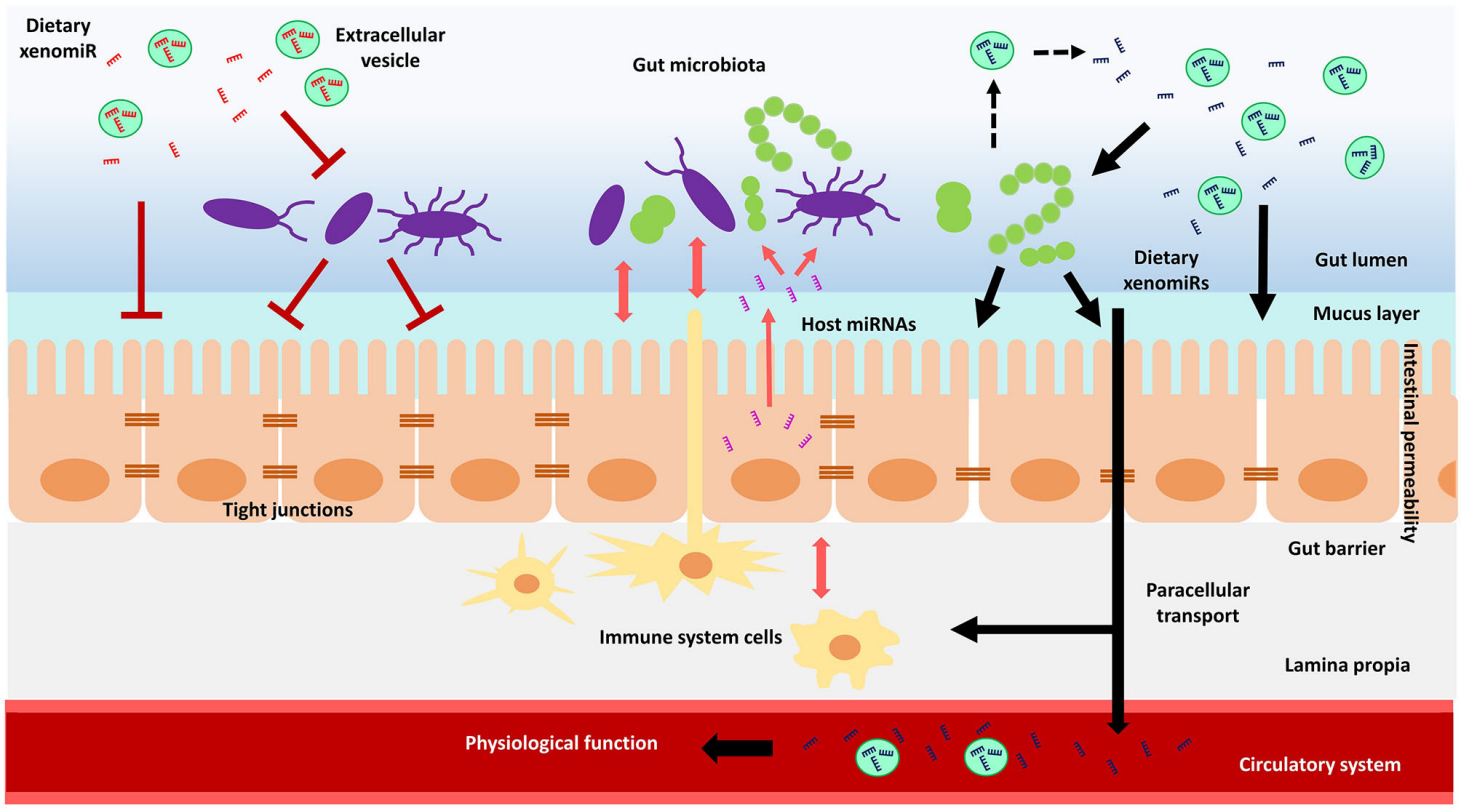
Schematic model of the hypothetical interactions of dietary plant xenomiRs with mammalian cells in the gastrointestinal tract. Gut microbiota could have an impact on intestinal barrier integrity and function, which in turn could modulate gut microbiota through molecules such as miRNAs. There could be also a crosstalk of immune system with both, intestinal barrier and gut microbiota. A certain xenomiR might promote or hinder its absorption depending on its effect on intestinal permeability, which might rely on the crosstalk between gut microbiota, intestinal epithelial cells and immune system. Gut microbiota might also be involved in the release of xenomiRs encapsulated in extracellular vesicles. The increase in intestinal permeability through, for example, altered tight junctions, might enhance xenomiR paracellular transport across the gut barrier, to eventually reach the circulatory system and potentially exert a physiological function in mammal tissues and organs.

Notably, diet is a key driver in establishing gut microbial communities composition, influencing microbiome diversity and ultimately determining inter-individual microbiome variability (204–207). As part of diet, plant xenomiRs are rising as promising candidates for the modulation of gut microbiota composition, intestinal immune system and intestinal epithelial cells functions, which in turn might affect intestinal barrier permeability and xenomiR absorption efficiency. Nonetheless, further investigation is mandatory to evaluate the potential role of plant xenomiRs on their own absorption and the bioavailability of other bioactive molecules, by shaping microbiota composition, microbe-derived metabolite production, intestinal epithelial cell and immune system function. A schematic representation of the potential interactions and mechanisms of action of plant xenomiRs with mammalian cells within the gastrointestinal tract is depicted in Figure 1.

It has been proposed that a large variety of microenvironments would exist throughout the gastrointestinal tract and gut microbes would thrive in those niches that best suit their nutritional and environmental requirements, leading to the establishment of diverse microbial communities along the gastrointestinal tract (208). Therefore, diet would be a crucial factor in determining variability of gut microbiota composition between individuals and, within an individual, in establishing different configurations of microbial communities throughout the intestine. In this context, recent studies have suggested that plant miRNAs could contribute to the dietary effect on gut microbiota community's assembly. Thus, Teng et al. proposed that ginger exosome-like nanoparticles small RNAs (and maybe other dietary small RNAs) could be involved in the spatial gut microbiota niche partitioning and in the selection of the bacteria that would be located near the intestinal epithelium (125). This finding provides a new avenue for further studying the following hypotheses:

1. If as a widespread property, plant xenomiRs could create different microenvironments throughout the gastrointestinal tract based on their location specificity and the bacteria they affect (84). This feature would promote specific bacterial selection in the different niches throughout the gastrointestinal tract, and therefore in determining the configuration of diverse microbial communities along de intestine and within different “microbial” layers.
2. If the hypothetical establishment of specific configurations of gut microbe communities and emergence of adaptive gut microbiota changes induced by xenomiRs, could have a direct impact on intestinal barrier integrity and function. Additionally, it should be explored if specific xenomiR-containing eating patterns could affect intestinal integrity and function and thus, determine the bioavailability of bioactive compounds.

Of note, along with proposed modulation of intestinal permeability mediated by the crosstalk between xenomiRs and gut microbiota, immune system and/or intestinal epithelial cells, other factors that would contribute to determine xenomiR bioavailability are the following:

1. Type and amount of xenomiR intake (24).
2. Acute/chronic (or short/long term) specific xenomiR intake (64).
3. XenomiR sequence-dependent absorption (79).
4. XenomiR association with uptake-facilitating molecules (65).
5. Extracellular vesicle transportation stability (83).
6. Gut microbiota hypothetical mediated-variations in the bioaccessibility of encapsulated xenomiRs (through the ability to hydrolyse envelope lipids of miRNA-containing plant extracellular vesicles and/or cellulose, hemicellulose and pectin fibers).

**Figure 2:**
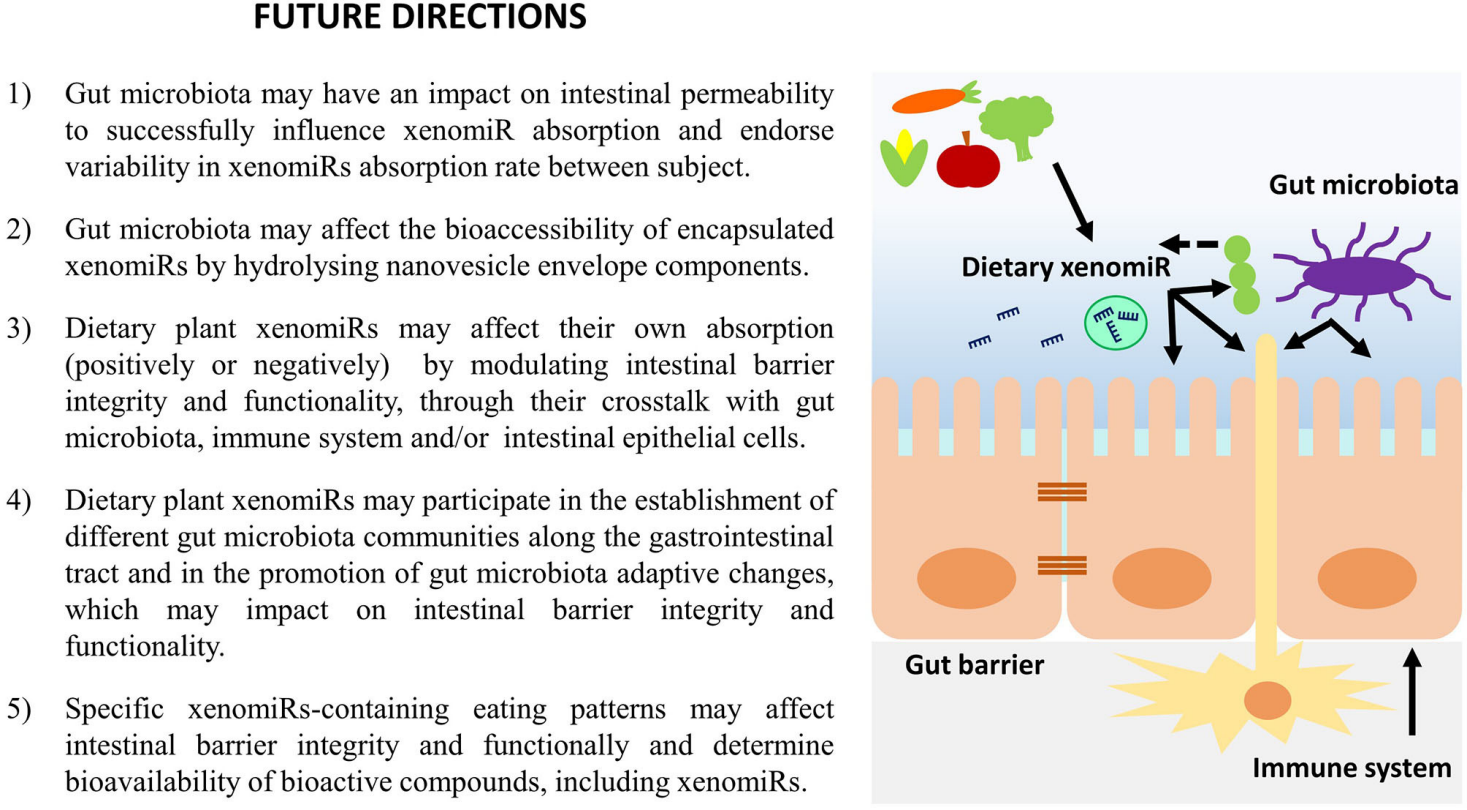
Hypothesis for the effect of dietary xenomiRs on gut microbiota, intestinal immune system and gastrointestinal barrier integrity and functionality.

A summary of the most important hypothesis concerning the effect of dietary plant xenomiRs on gut microbiota, intestinal immune system and gastrointestinal barrier presented within this work are summarized in Figure 2.

In conclusion, although it is well-established that gut microbiota plays an important role in determining the integrity and functionality of intestinal barrier, more studies are needed to verify the direct impact of gut microbiota-intestinal permeability modulation on xenomiR absorption efficiency, as well as the role of xenomiRs shaping this process. A better understanding of the interactions of xenomiRs with gut microbiota, immune system and intestinal epithelial cells, in the context of intestinal barrier modulation, could provide new insights on the mechanisms underlying variability of xenomiR intestinal absorption and emerge as new explanatory factors of the discrepancies regarding the hypothesis of xenomiR detection in animals.

## Author Contributions

ED-S, SL-C, and FM contributed to the ideas and hypotheses presented in the manuscript. ED-S wrote the paper, which was revised and edited by SL-C, FM, PA, JR-B, and JM. All the authors approved the final version.

## Conflict of Interest

The authors declare that the research was conducted in the absence of any commercial or financial relationships that could be construed as a potential conflict of interest.
